# Numbers are not the whole story: a qualitative exploration of barriers and facilitators to increased physical activity in a primary care based walking intervention

**DOI:** 10.1186/1471-2458-14-1272

**Published:** 2014-12-15

**Authors:** Rebecca Normansell, Jaime Smith, Christina Victor, Derek G Cook, Sally Kerry, Steve Iliffe, Michael Ussher, Julia Fox-Rushby, Peter Whincup, Tess Harris

**Affiliations:** Population Health Research Institute, St George’s University of London, London, SW17 ORE UK; Gerontology and Health Services Research Unit, College of Health and Life Sciences, Brunel University, London, UB8 3PH UK; Pragmatic Clinical Trials Unit, Queen Mary’s University of London, London, E12AT UK; Research Department of Primary Care & Population Health, University College, London, NW3 2PF UK; Health Economics Research Group, Brunel University, London, UB83PH UK

**Keywords:** Qualitative research, Physical activity, Pedometer intervention, Walking, Primary care, Older adults

## Abstract

**Background:**

The majority of mid-life and older adults in the UK are not achieving recommended physical activity levels and inactivity is associated with many health problems. Walking is a safe, appropriate exercise. The PACE-UP trial sought to increase walking through the structured use of a pedometer and handbook, with and without support from a practice nurse trained in behaviour change techniques (BCTs). Understanding barriers and facilitators to engagement with a primary care based physical activity intervention is essential for future trials and programmes.

**Methods:**

We conducted semi-structured telephone interviews using a topic guide with purposive samples of participants who did and did not increase their walking from both intervention groups. Interviews were audio-recorded, transcribed and coded independently by researchers prior to performing a thematic analysis. Responsiveness to the specific BCTs used was also analysed.

**Results:**

Forty-three trial participants were interviewed in early 2014. Almost all felt they had benefitted, irrespective of their change in step-count, and that primary care was an appropriate setting.

Important facilitators included a desire for a healthy lifestyle, improved physical health, enjoyment of walking in the local environment, having a flexible routine allowing for an increase in walking, appropriate self and external monitoring and support from others.

Important barriers included physical health problems, an inflexible routine, work and other commitments, the weather and a mistrust of the monitoring equipment.

BCTs that were reported to have the most impact included: providing information about behaviour-health link; prompting self-monitoring and review of goals and outcomes; providing feedback; providing specific information about how to increase walking; planning social support/change; and relapse prevention. Rewards were unhelpful.

**Conclusions:**

Despite our expectation that there would be a difference between the experiences of those who did and did not objectively increase their walking, we found that most participants considered themselves to have succeeded in the trial and benefitted from taking part. Barriers and facilitators were similar across demographic groups and trial outcomes. Findings indicated several BCTs on which PA trial and programme planners could focus efforts with the expectation of greatest impact as well as strong support for primary care as an appropriate venue.

**Trial registration:**

ISRCTN98538934.

## Background

Approximately 90% of all National Health Service (NHS) contacts in England take place in general practice [1] and thus primary care is ideally situated to deliver physical activity (PA) interventions, and has indeed been the setting for a number of trials [2–5]. Such trials have been subject to systematic review, including reviews of primary care based interventions and exercise-on-referral schemes [6–8]. Some trials have focussed on increasing walking, both in primary care [9] and in community settings [10].

Walking is an appropriate exercise for almost everyone and the most common exercise among older adults [11]. However, data indicates that, using objective measures, <10% of men and women aged 16 and over were achieving recommended PA levels in 2008, despite between 29% of women and 39% of men reporting doing so [12]. This suggests an important discrepancy between what many people perceive they are achieving in terms of PA and their actual levels. Current UK PA guidelines for adults and older adults recommend at least 150 minutes moderately intensive PA weekly, or 75 minutes vigorous PA weekly, both in at least ten minute bouts [13]. This guidance and promotion of PA is reflected in the key priorities of Public Health England [14].

PACE-UP is a 3-arm randomised controlled trial (RCT) aiming to increase walking in 45–75 year old inactive primary care patients through the use of pedometers, with and without additional support from a practice nurse. In the pedometer by post group, participants were sent a pedometer, PA handbook and diary with the intention that they would complete the intervention independently. In the nurse support group, participants were given the same equipment and materials but in the context of a practice nurse PA consultation. Participants in this group met with the nurse a further two times over the course of the three month intervention. The trial’s control group were asked to continue with their usual level of activity. Further detail of the PA intervention is presented in (Table 1).

Nursing staff were trained in the use of behaviour change techniques (BCTs) by experienced trainers and participant materials, received by both intervention groups, were prepared in line with BCT principles. BCTs used adhered to the CALO-RE taxonomy, developed to identify BCTs used in PA or healthy eating interventions (Table 2, [15]). This is widely considered to be the leading taxonomy for BCTs and the rationale for its use is described in detail in the trial protocol [16]. The theoretical basis for the intervention does not rely on one specific model but, in line with the NHS Health Trainers’ Handbook [17] and NICE guidance [18], recognises that a number of difference approaches may be useful.

**Table 1:** **Summary of the PACE-UP walking programme**

Week of PACE-UP walking programme	Target number of steps
1-2	Add in 1500 steps on 3 or more days per week
3-4	Add in 1500 steps on 5 or more days per week
5-6	Add in 3000 steps on 3 or more days per week
7-12	Add in 3000 steps on 5 or more days per week
Remember 1500 steps equals about 15 minutes of walking and 3000 steps equals about 30 minutes of walking	

The plan is to start from where **you** are currently and gradually increase the amount you walk over 12 weeks.

Use the pedometer to record the number of steps you do each day and write them in your PACE-UP diary.

Participants were recruited from seven South-West London (UK) general practices, representing diverse socio-economic and ethnic groups. Baseline step count was ascertained by accelerometry and a blinded pedometer. Participants were followed up at 3 and 12 months. The main outcome was an increase in average daily step-count, measured by accelerometry at 12 months [16].

There is a wealth of literature that attempts to address the factors that affect adherence to a PA programme and the barriers and facilitators for increased activity [19–27]. Some call for further research into the link between adherence and theory-based PA interventions [21, 24, 26] while others call for more objectives measures of PA and its determinants [19] and more intervention studies [23]. Several studies report unique barriers in older adults and the importance of tailoring interventions to overcome these barriers [19, 27]. Some intervention studies have included a structured qualitative analysis [9, 28, 29], albeit with relatively small numbers (n <25) compared with the PACE-UP trial. In our study, we attempt to address some of the research needs identified by performing a structured qualitative analysis of patient experience in a theory-based PA trial that recruited both mid-life and older adults. To our knowledge, this is the largest primary care based walking intervention with a structured qualitative analysis.

While objective accelerometry data can tell us whether an intervention worked for an individual, it offers little insight into the reasons for that individual’s experience. Understanding why and how an intervention worked (or did not work) is essential for the success of future trials and PA programmes. Qualitative interviews allow the in depth exploration of the intervention experience with participants and enable us to discover how different elements of the BCTs were received.

### Aim

In-depth exploration of the experiences of samples of participants from both intervention groups who increased their step-count and who did not increase their step-count from the PACE-UP primary care PA trial.

## Methods

### Sampling and recruitment

Consent to take part in a follow-up telephone interview was gained from all participants at the time of recruitment into the trial. In January 2014 a spreadsheet of participants who had completed 12 month follow-up and given consent to be interviewed was produced by the trial statistician, blinded to group allocation. The qualitative researchers were un-blinded and those who had been allocated to the control group were excluded as they had not received an intervention. We selected groups of participants who had and had not increased their step count at 12 months, from the two intervention groups; approximately half from the nurse-led pedometer group and half from the pedometer by post group. For the purposes of the qualitative study, we defined an increase pragmatically as >/=200 steps/day. Participants whose step-count deceased or failed to increase by >/=200 steps/day were regarded as having no increase. This threshold was agreed after discussion between authors.

We used the demographic information on the spreadsheet to ensure our study group represented a range of ages (approximately half 45–59 years old and half 60–75 years old), at least 5 participants from each of the 6 practices with 12 month data, both genders (to reflect the approximately two-thirds females in the study), a range of ethnicities, and to include some participants who had taken part in the study as a couple. We purposively targeted potential participants from demographic groups under-represented in our main sample to ensure we explored the widest range of views possible.

A second search was conducted for patients completing 12 month follow-up in March 2014 to ensure we sampled participants completing their intervention at a different time of year and who were recruited at a time when the trial was more established.

We planned to interview at least 40 participants to ensure we sampled a minimum of 10 people from each intervention group who had both increased and not increased their PA, in line with our strategy. After reaching 40, we planned to continue recruitment until we had achieved thematic saturation and a broad demographic balance. As part of our strategy to ensure the credibility of our findings the researchers regularly met throughout the recruitment to ensure the sampling was progressing as planned and to discuss emerging themes.

### Interview methodology

We conducted semi-structured audio-recorded telephone interviews with participants using an interview schedule [Appendix 1]. Those selected for telephone interview were reminded of their initial consent to be interviewed and if they were happy to go ahead, their consent was also sought to be audio-recorded. The interview schedule was modelled on that used during a preceding PA trial [30] and refined by discussion between authors and piloting prior to recruitment. The schedule was adapted slightly after the first 15 interviews, following discussion between co-authors, to ensure better understanding of the questions and also to probe participants’ memories further with regard to meeting with the practice nurse, as we found that few participants were spontaneously volunteering specific details.

All interviews were promptly transcribed verbatim and circulated to other members of the author team to ensure consistency between interviewers, to manage any problems as they arose and to identify when thematic saturation had been achieved. In order to assess the response to the invitation to be interviewed, we kept a detailed record of all participants who we attempted to contact, who agreed, who refused and who could not be contacted. Interviewers attempted contact up to a maximum of three times and at different times of day to ensure we captured views from those who might be unavailable during working hours.

### Analysis

To support the credibility of our analysis all transcripts were read and re-read by RN, JS, CV and TH and codes were assigned independently. Discrepancies were resolved by discussion and peer debriefing and codes were then grouped into themes. These codes and themes were further refined by discussion between RN and JS following established standards [31, 32] to produce broader themes, each encompassing several sub-themes.

The use of specific theoretically-informed BCTs was an important element of the trial. Practice nurses delivering the intervention had initial training in BCTs by experienced trainers, which continued at scheduled intervals throughout the intervention. BCTs were also incorporated into the participant handbook that both intervention groups received. The BCTs used are summarised in Table 2[15]. As we were interested in understanding which of these techniques had been of most use to participants, we performed an additional analysis of the data to specifically draw out themes relevant to each of these techniques.

### Ethics

The trial was reviewed and approved by the London Research Ethics Committee (Hampstead) (12/LO/0219). National Health Service Research and Development approval was given initially by Primary Care Trusts and then by Clinical Commissioning Groups in South-West London to cover all the practice sites.

## Results

Between February and April 2014 we interviewed 43 trial participants by telephone. We attempted to contact 96 trial participants and successfully made contact with 44, of whom 1 declined to be interviewed, citing a recent bereavement. Interview duration ranged from 9–44 minutes, with an average duration of 21 minutes. Eight were conducted by JS with the remainder by RN. Thematic saturation was achieved prior to completing 40 interviews but, as planned, we continued in order to ensure a demographically balanced sample.

### Interview participants’ characteristics [Tables 3 and 4]

**Table 2:** **Behaviour change techniques used in the PACE-UP trial by practice nurses and in participant handbook and diary**

Behaviour change technique	Number from Michie’s taxonomy [15]
Provide general information on behaviour-health link	1
Provide information on consequences to individual	2
Provide normative information about others’ behaviour	4
Action planning	7
Barrier identification	8
Set graded tasks	9
Prompt review of behavioural goals	10
Prompt review of outcome goals	11
Prompt rewards contingent on effort	12
Prompt rewards contingent on successful behaviour	13
Prompt self-monitoring of behaviour	16
Prompting self-monitoring of behavioural outcome	17
Prompting focus on past success	18
Provide feedback on performance	19
Provide information on when and where to perform the behaviour	20
Provide instructions on how to perform the behaviour	21
Teach to use prompts/cues	23
Prompt practice	26
Plan social support/social change	29
Relapse prevention/coping planning	35
Stress management/emotional control training	36
Motivational interviewing	37

Interview participants represented those who had increased and not increased their walking from all 6 practices, a range of ages and ethnicities, men and women, from both intervention groups and included some who had taken part as a couple.

### Thematic and BCT analysis of interview responses (Tables 5 and 6)

**Table 3:** **Summary of interview participant characteristics (n = 43)**

Characteristic		Participant n
**Age**	45-59 years	20
	60-75 years	23
**Gender**	Male	14
	Female	29
**Ethnicity**	White British	29
	Any other white background	5
	Black African	2
	Black Caribbean	2
	White and black Caribbean	1
	Bangladeshi	1
	White and Asian	1
	Indian	1
	Chinese	1
**Primary care practice**	1	7
	2	7
	3	7
	4	7
	5	7
	6	8
**Intervention group**	Nurse and pedometer	21
	Pedometer by post	22
**Step count outcome**	Increase	20
	No increase	23
**Group + step count outcome**	Increase + nurse/pedometer	10
	Increase + pedometer by post	10
	No increase + nurse/pedometer	11
	No increase + pedometer by post	12
**Recruited as a couple?**	Yes	7
	No	36

**Table 4:** **Interview participant details**

ID No.	Practice No.	Male/Female	Couple?	Self-reported ethnicity	Group	No. of nurse appts attended (out of 3)	Age	Change in average steps/day from baseline ^1^	Health problems divulged during interview
1	1	Female	No	Any other White background	Nurse	3	48	+1697	Nil
2	1	Male	No	White British	Nurse	3	45	+113	Nil
3	1	Male	No	White British	Pedometer	N/A	53	+3708	Yes – preceding the trial
4	1	Male	No	Bangladeshi	Pedometer	N/A	52	−234	Nil
5	1	Female	No	White British	Pedometer	N/A	57	+1718	Nil
6	1	Female	No	White British	Pedometer	N/A	51	−2141	Yes– preceding the trial and during the trial
7	1	Female	No	White British	Pedometer	N/A	60	−1808	Yes – preceding the trial
8	2	Female	No	White British	Pedometer	N/A	65	−1781	Nil
9	2	Female	No	Black Caribbean	Pedometer	N/A	69	+243	Yes – preceding the trial and during the trial
10	2	Male	No	Black African	Nurse	2	64	−1920	Yes – preceding the trial
11	2	Male	No	White British	Pedometer	N/A	70	+1543	Yes – during the trial
12	2	Female	No	White and Black Caribbean	Nurse	3	66	+1211	Yes – preceding the trial
13	2	Female	No	White British	Pedometer	N/A	66	−446	Yes – preceding the trial and during the trial
14	2	Female	No	Any other White background	Nurse	2	49	+4756	Nil
15	3	Female	No	Any other White background	Nurse	3	49	−1097	Yes – during the trial
16	3	Female	No	White British	Nurse	3	47	+1573	Nil
17	3	Female	No	Any other White background	Pedometer	N/A	66	−1027	Yes – preceding the trial
18	3	Female	Yes	White British	Nurse		62	−2836	Nil
19	3	Female	No	White British	Pedometer	N/A	66	−1797	Yes – preceding the trial
20	3	Male	Yes	White British	Nurse	3	52	+3924	Yes – preceding the trial
21	3	Female	No	Black African	Nurse	3	47	+2962	Yes – preceding the trial
22	4	Male	No	White British	Nurse	3	63	−2652	Yes – preceding the trial and during the trial
23	4	Female	Yes	White British	Pedometer	N/A	64	+226	Yes – preceding the trial and during the trial
24	4	Female	Yes	Any other White background	Pedometer	N/A	50	+1031	Yes – preceding the trial
25	4	Male	No	White British	Pedometer	N/A	67	−955	Yes – preceding the trial
26	4	Female	No	White British	Nurse	3	65	−2013	Yes – preceding the trial
27	4	Male	No	White and Asian	Pedometer	N/A	61	−611	Yes – during the trial
28	4	Female	No	Chinese	Nurse	3	72	+4062	Yes – preceding the trial
29	5	Male	No	White British	Nurse	3	59	−493	Yes – during the trial
30	5	Female	No	White British	Nurse	3	51	+3269	Yes – preceding the trial
31	5	Male	Yes	White British	Pedometer	N/A	59	−756	Nil
32	5	Female	No	White British	Nurse	3	63	+1966	Yes – during the trial
33	5	Female	No	White British	Nurse	3	49	−746	Nil
34	5	Female	No	Black Caribbean	Pedometer	N/A	73	+403	Yes – preceding the trial
35	5	Female	No	White British	Nurse	3	64	+2100	Yes – preceding the trial
36	6	Female	No	White British	Pedometer	N/A	64	+1639	Nil
37	6	Female	No	Indian	Pedometer	N/A	51	−1720	Nil
38	6	Female	Yes	White British	Pedometer	N/A	59	+539	Nil
39	6	Female	No	White British	Nurse	2	61	−1425	Nil
40	6	Male	Yes	White British	Nurse	3	48	−3826	Yes – during the trial
41	6	Male	No	White British	Nurse	2	65	−43	Yes – during the trial
42	6	Male	No	White British	Pedometer	N/A	72	−2133	Nil
43	6	Female	No	White British	Pedometer	N/A	48	+2253	Nil

1. For the purposes of the qualitative study, we defined an increase in step count as >/=200 steps/day. Participants whose step count deceased or failed to increase by >/=200 steps/day were regarded as having no increase.

**Table 5:** **Summary of thematic and behaviour change technique analysis**

Themes	Sub-themes	Behaviour change techniques [15]
Healthy Lifestyle	Feeling fitter	Providing general information on behaviour-health link
	Sleep	Providing information on consequences to the individual
	Weight loss	
	Awareness of walking	
Physical Health	Specific health problems	
	Pain	
Environment	Location of appointments	
	Weather/season/climate	
	Locality for walking	
	Work	
	Pets	
Routine	Fixed routine	
	Fluctuating routine	
	New routine	
Monitoring	Targets	Set graded tasks
	Self-efficacy/self-monitoring	Prompt review of behavioural goals
	External monitoring and feedback	Prompt review of outcome goals
	Equipment used for monitoring	Prompt rewards contingent on effort
		Prompt self-monitoring of behaviour
		Prompt self-monitoring of behavioural outcomes
		Provide feedback on performance
Social Perspectives	Peer support/encouragement	Barrier identification
	Meeting others	Provide information on when and where to perform behaviour
	Impact on others	Provide instruction on how to perform behaviour
		Relapse prevention/coping planning
		Providing normative information about others’ behaviour
		Plan social support/social change

**Table 6:** **Themes and sub-themes with supporting quotes**

Theme	Sub-theme	Evidence
**Healthy lifestyle**	Feeling fitter – both as a goal and an outcome	[via translator] She said that before the trial she feels so tired and she sometimes was worrying because she had… granddaughter but now she feel good and she can enjoy going … she can enjoy with her granddaughter in the park or sometimes around they walk. (IDN14)
		I mean you know I think it has made a difference and it does make me think about where I’m walking and how far I’m walking and the health benefits, so yes, I think it was a really good thing and I think it would be a really great thing if more people could actually have the support to do something like that. (IDN30)
	Sleep	..when I go walking, it helps and I sleep better as well when I go walking, and come back, I have a better night’s sleep, and all aches and pain disappear. (IDN34)
	Weight loss – both as a goal and an outcome	And since the beginning of the uhh trial I’ve lost just over a stone in weight… I feel fitter now than I have done for years. (IDN11)
		I want to lose some weight and make myself healthy, just healthy, you know. (IDN28)
	Awareness of walking as part of a healthy lifestyle	I think you know umm getting people to increase their step count is a great idea because it’s an easy way of exercising and you know and the health benefits are good, so I’m very much supportive of the aims of the study…You become more aware of the benefits of walking. (IDN38)
**Physical health**	Specific health problems – worsening and improving	I do have arthritis…and I had a bad spell during the middle where I’d done something to my knee..it was a pulled muscle or something.. So I struggled to walk too far. (IDN3)
		I was having some physio, that was in January, and with the combination of the physio and the walking, and I don’t know whether it was just because of swinging my arms and there was more movement, but I think it helped with that as well, and even the physio said he thought it had helped. (IDN30)
	Pain	..now what I have noticed is that, if I’m in to exercise regimes, that are sustained and regular, I feel much less of that pain. (IDN10)
		I had suffering a back ache, you know, and leg pain and things like that, but when I go walking, it helps. (IDN34)
**Environment**	Primary care as the location	Yes, that was good, because obviously it was very near home so it was ideal. (IDN15)
		Yes, I mean I think it’s … it’s good being able to do that. It’s fairly local for me, you know, easy to get to. Much easier than if I had to go to a hospital you know so, yes, that was easy. (IDN30)
	Weather/season/climate	I enjoyed the fresh air and the exercise and I felt better for it. (IDN2)
		In winter it dies down a little bit, anyway, you just want to get out of cold and rain and you just make it home asap and you’re just less active. So there’s a seasonal thing definitely, you know, so if it’s summer, you are out and about all day anyway. (IDN1)
		Well obviously it’s much nicer to go for a walk in the spring or the summer or the autumn, so if you do hand these things out in the winter, people don’t think I’ll go for a walk, oh no, God it’s cold out there, it’s raining. (IDN13)
	Locality for walking	..it was really nice to start going out for little walks in the neighbourhood and finding places, little parks, and little cafes that I hadn’t been to before, so that was another positive thing. (IDN6)
		I think I’m lucky. I’ve got a small park, close to me and so whereas I may have walked to the shops, I would do three circuits or so of that and then go on to the shops. (IDN35)
	Work	It depends what I’m doing because if I’m at work, then I’m obviously not as active because I’m in a small confined space and I just walk up and down when I need to sort of thing. (IDN7)
		An awful lot of what I maybe can do now is likely sedentary involvement. I’m doing online work which really confines me to sitting you know and doing a lot of work on the computer. And I have to make a conscious effort both for going out and doing the necessary walking. (IDN10)
	Pets	I have moved in to a place with three dogs, can’t get any better, so I walk more I think maybe now. (IDN1)
		I know this week I haven’t got the dog and we’ve been really busy and I’ve been much more car bound so I would dread to think what my steps were this week. And that’s why I think, having a dog, is fantastic, because you have to go out anyway. (IDN33)
**Routine**	Fixed routine – unable to increase walking	I didn’t find it physically that difficult to actually reach it, but just very time consuming and I just with working I found that I..just didn’t have the sort of time or enthusiasm for doing that. (IDN2)
		The walking is because I do the same all the time you know. Every day is my routine walk you know, going to work... Because you know I do it every day, the walking in this way, I don’t need any help for anyone. (IDN4)
		I’ve had a very bad year, work-wise, well I’m working under a lot of pressure, and time to go and do things wasn’t really there. (IDN22)
		I mean there is a limit to the amount of exercise … time I can spend exercising in a day. (IDN31)
	Fluctuating routine – sometimes allowing for walking, sometimes not	Well I don’t think I ever reached the actual total targets. I did on odd days. But you know it was all depended on my umm … commitment because, if I was you know … out somewhere and that, I couldn’t do the walking, it was alright when I was just at home and I could just walk round the roads and get my steps up, do you see what I mean? (IDN19)
		I mean, to me, it very much depends what’s going on in my life. I have a dog, I have a husband who at the beginning of last year changed his job so he’s at home much more, which has changed my routine enormously. And I … that has really affected my exercising. But I mean on a daily basis I’m very busy. So I’m probably good on steps, yes. But when I don’t have the dog, which I haven’t done for the last few weeks, then it goes down enormously. (IDN33)
	New/flexible routine	…a really good outcome, I’ve just thought, we hardly ever use the car. Only when I do a big shop. Or we’re going to visit someone, we hardly ever use the car. That’s a real plus. (IDN18)
		Yes, everyone in my house now, we don’t drive to the shops, we all walk to the shops…Because I’m the one who drives so I just say I’m not driving, I’m walking.. so we end up walking, so that’s the influence, it was easier for me just to jump in to the car, now I have to think twice do I really have to? (IDN21)
		I did try to increase it to five times a week, and I’ve certainly kept that up now, so for instance, I’ve been to the gym this morning. Tomorrow I’ll make sure that I walk in to town and walk back again, so that at least five times a week, doing at least minimum of 30 minutes, but three times a week it’s an hour or more. I think the PACE-UP programme has kind of set me off on a new regime of keeping fit. (IDN39)
**Monitoring**	Targets	It seemed like a challenge and I think it’s one of those things as well that, when you’re … you’re given a challenge, umm… I think I started slipping once it had finished and that is because I didn’t have that challenge there anymore. (IDN30)
		It was very positive, it was very positive in that every time I managed to achieve and go beyond it was … there was a real boost. (IDN37)
		Sometimes it was a bit demoralising because you kind of thought, oh, I can’t possibly do that number of steps, you felt like you were never reaching your goal, so that was probably a bit … it wasn’t de-motivating but it’s a slightly demoralising to think you’re not reaching your targets isn’t it, so I think maybe the targets … should consider the targets you are setting people with a time or maybe I just should walk faster or something. (IDN38)
	Self-efficacy/self-monitoring	Well part of it, part of it, but I myself pushed myself more as well because I didn’t go to the nurse that often, then I was on my own, so I had to be dependable on my own strength and walk. (IDN12)
		What would she say, walk a bit quicker, eat a bit less? It’s common sense I knew in the first place…I don’t think a nurse or anybody else telling me what I should do would really make much difference, quite honestly. (IDN23)
		I’m quite a self-motivated person so I don’t … I think if I’ve agreed to do something, then I will try and achieve that target, whether somebody tells me face to face or by post, so I think it’s dependent on the individuals maybe, individual choice. (IDN38)
	External monitoring and feedback	So that even when I feel like I am giving up, it’s more like I am thinking, but no, somebody will be watching me! (IDN21)
		..it’s having someone to get some feedback from because then you know you aren’t doing things in vain. (IDN22)
		There’s nothing like the fact that you know you’re going to meeting someone and talk about it to make you do it, you know, when I was at work, we used to have this thing about, as I said, you’d rather do things they enjoy or things they are checked up on… It’s basically the routine of being checked up on by someone else and being part of a group of people. (IDN29)
		Yes, it would have been incredibly useful, yes, it would have been. It would have been, for two-fold, one to get some sort of feedback, the other one just to sustain that level of interest in the programme. Purely because you’re having to … you’re being monitored and you’re having to respond to certain key stones, key points, and I think by the very nature of human beings, when we’re being monitored, we do things. (IDN37)
	Equipment used for monitoring	Oh I did always look. Actually it was quite … and if I had had a low … the weekend tends to be lower, it did just jog my … yes, it made me think, okay, fine, I must do a little bit more. (IDN33)
		I wasn’t too sure about its accuracy because sometimes I would do a similar route and it would give a different reading, quite a large different reading. (IDN11)
		Sometimes it [the pedometer] didn’t pick things up, I used to get annoyed because it wouldn’t pick things up in the gym, I’d think oh, I’ve just done about 2,000 strides, and I’ve looked down and think, ooh, you didn’t get any of that. (IDN43)
**Social perspectives**	Peer support/encouragement (including positive thoughts about group setting)	It’s a bit like talking to people who are in the same situation as yourself, you know, because no-one understands the issues of someone better than someone who is in that situation themselves. I’m not saying that there are other ways of coaching and supporting people, but I’m just thinking of the things that tend to change peoples’ behaviour. (IDN29)
		I think sometimes it can be helpful to talk in a group because you might have an idea that somebody else hasn’t thought of and vice versa. (IDN30)
		I think you could sort of encourage each other, yes, it’s more fun doing stuff with other people really isn’t it. (IDN43)
	Meeting others (including positive thoughts about group setting)	Sometimes I’m walking, and I meet somebody, and they will stop and talk to me and ask me the reason why. And I’ll say to them, walk with me, and we would talk, you know, and that I think that was very good. (IDN9)
		Sometimes it’s a way of getting to meet other people in your area that you could actually, I suppose say, shall we all do this together and something like that, you never know. (IDN18)
	Impact on others	I’ve got a son of 16, its made me kind of like if he says can I have lift or something like that, I say, and because I do a lot of driving with my job, the last thing I want to do is get in the car, I say, no, but I’ll walk up there with you, and sometimes he says no thanks, I’ll walk on my own, but its made me more … I think its increased his walking as well because of … because of this. I think it has been really good from that point of view, yes. (IDN16)
		I think we’ve now managed to encourage our sister-in-law to start walking. She’s now been going out walking because she can see the benefits of how we’ve got on, you know, because we go for quite long brisk walks and we don’t get out of breath or anything, so umm she sort of thought, oh, well I’ll start my walking now, because she’s quite overweight. (IDN20)

We had anticipated that there would be differences in the responses given by those who had and those who had not increased their walking. However, we found that there was substantial overlap between the responses of these two groups, with many participants for whom the trial had not been a ‘quantitative success’ still feeling they had gained a great deal from participating. For this reason, we have not attempted to analyse the interviews according to those who had increased or not increased their activity, but rather drawn themes from their responses as a whole.

In total, we found 152 discrete examples of BCTs being alluded to by participants, 54 in the pedometer by post group and 98 in the nurse group. The only BCT domain in which the pedometer by post group references dominated was in those related to self-monitoring. Responsiveness to the BCTs used in the handbook, diary and nurse consultations is discussed alongside the main thematic analysis.

### Healthy lifestyle

The importance of a healthy lifestyle emerged both as a motivator for participation and/or an outcome of taking part in the trial. Participants discussed the concepts of feeling fitter, improved sleep, weight loss and their awareness of walking as part of a healthy lifestyle within this theme.

The concept of feeling generally fitter was important and was discussed in terms of living longer for grandchildren, being able to enjoy life more fully and keeping more active into older age:

*“I mean I think the walking does make a difference, it certainly does… I don’t do jogging, but walking certainly does, because it keeps you moving, keeps you fitter.”***(IDN22, male, aged 63, white British, nurse group, no increase).**

*“..it’s just a straightforward correlation between walking more and being generally fitter in old age, sort of makes sense.”***(IDN 29, male, aged 59, white British, nurse group, no increase).**

Examples of more specific motivations were the benefits of exercising for sleep and weight management. Some lost weight during the trial and some expressed disappointment that they did not lose more weight:

**“***I sleep better as well when I go walking, and come back, I have a better night’s sleep”***(IDN34, female, aged 73, black Caribbean, pedometer only group, increase).**

*“I consciously knew I was overweight when I started it and, at the end of it, I think I’d probably shed over a stone, so … seven kilos, six or seven kilos.”***(IDN3, male, aged 53, white British, pedometer only group, increase).**

*“I was hoping I might lose some weight doing the trial but I didn’t.”***(IDN7, female, aged 60, white British, pedometer only group, no increase).**

Participants expressed great awareness of walking as part of a healthy lifestyle and many felt that this awareness had been enhanced by their experience of taking part in the trial:

*“We want to keep fit… I think it’s made us extremely aware and looking at friends that are not half as active and that have complaints, touch wood, we’re doing well.”***(IDN18, female, aged 62, white British, nurse group, no increase).**

Linked in with the healthy lifestyle theme are techniques 1 and 2 from Michie’staxonomy [15]; providing general information on behaviour-health link and providing information on consequences to the individual. We found substantial evidence that participants valued the provision of this information both from the practice nurse and from the trial literature:

*“I thought it was very good and it did bring to mind how much exercise or not one is doing, so from that aspect, I found it umm … very useful for me.”***(IDN39, female, aged 61, white British, nurse group, no increase).**

*“You become more aware of the benefits of walking and try to walk places rather than drive and try to build in time to walk because obviously it takes longer to walk somewhere than drive, or get the bus or whatever. I think you try … you become more in tune with your health, so I’ve tried to lose some weight and things like that alongside the walking.”***(IDN38, female, aged 59, white British, pedometer only group, increase).**

### Physical health

Physical health was discussed by participants, in terms of specific health conditions as both being a barrier to increasing their activity and as a motivation to increase activity so as to improve physical health. Of note, 37% mentioned at least one health problem that preceded the trial, 14% mentioned a health problem that occurred for the first time during the trial and 12% mentioned both. Only 37% of participants did not discuss health problems during their interview. Most felt that walking was beneficial:

*“I think it makes it (arthritis) better… Because the more I walk the better I am. I’ve mainly got osteoarthritis in my hands, but sometimes my knees feel weak, but the walking doesn’t hinder me, it helps it because it keeps you more lubricated.”***(IDN26, female, aged 65, white British, nurse group, no increase).**

*“Because it’s good for your blood pressure, it’s good for high cholesterol, good for diabetes.”***(IDN9, female, aged 69, black Caribbean, pedometer only group, increase).**

However, a minority felt that attempting to increase their walking may have been detrimental:

*“And unfortunately… being part of the trial and trying to increase the level of activity was probably the worst thing I could have done. But we didn’t know that and I so wanted it to work but I’ve learned a lot about the illness* [myalgic encephalomyelitis, ME] *and know that I would have to increase it in tiny tiny steps.”***(IDN6, female, aged 51, pedometer only group, white British, no increase).**

*“I somehow I think I overdid it and injured my knee, which meant that I was limited in the amount of walking I could do.”***(IDN25, male, aged 67, white British, pedometer only group, no increase).**

Several participants specifically mentioned pain, and all felt that walking in fact reduced their symptoms:

*“I enjoyed it because it helps me to … makes my body… the pain in my knees and my shoulder. That helps… the more I go, the pain gradually decreased.”***(IDN9, female, aged 69, black Caribbean, pedometer only group, increase).**

### Environment

Many participants reflected on the environment in which they lived and the impact this had on their ability to increase their walking. This included the sub-themes of the primary care setting for the trial, the weather and season, the locality for walking, participants’ working environment and the influence of pets.

Primary care was universally thought to be an appropriate and convenient location to deliver this type of intervention:

*“Yes, yes, that’s easy for me. I walked there and walked back so it’s a nice sort of 15 minute walk.”***(IDN16, female, aged 47, white British, nurse group, increase).**

*“..you wouldn’t want someone to have to travel and people know how to get to their doctors don’t they?***(IDN 29, male, aged 59, white British, nurse group, no increase).**

The environment also had an impact on participants’ experience in terms of the weather. Some felt that dark, cold and wet weather was no hindrance, while others admitted that it probably affected their progress:

*“I don’t pay too much attention to what happens out there, as long as it’s not floods. The winter, you know, inclement as they tend to be, are not a barrier.”***(IDN10, male, aged 64, black African, nurse group, no increase).**

*“..if it involves going for a walk around* [the local area] *in the evening, in the rain, no, it’s not that appealing, I mean, if I’d been doing it in the summer, I would have found it easier if it was light in the evening and the weather was nice, I could have walked round the park or something, but that’s not really an option in the middle of winter”***(IDN2, male, aged 45, white British, nurse group, no increase).**

The locality for walking was also an important factor for some of our interviewees, with some reflecting how a pleasant walking environment facilitated their increase in activity while others discussed how less pleasant surroundings were a barrier:

*“If you have a park and things in the area, of course, I think that’s more motivational than if you live at Piccadilly Circus”***(IDN1, female, aged 48, any other white background, nurse group, increase).**

*“I think it also depends on where you live. I mean I’m lucky, I live near the common, so it’s really easy for me to just go out and walk, rather than just walking round the streets and things. And so it probably has to be related to the area … progress has to be related to where it is that you live.”***(IDN33, female, aged 49, white British, nurse group, no increase).**

Many of our participants were working in part or full time jobs and they perceived that this had a substantial impact on their ability to engage with the trial. Some found that they could build walking into their working day, while others felt this was simply not possible:

*“10,000 steps in a day is a lot of walking, but you know … in my previous job I would have been doing, on a regular basis, daily basis I’d have said, comfortably. In this job you know I have to … I don’t get up and walk around like I used to.”***(IDN3, male, aged 53, white British, pedometer only group, increase).**

*“..when I started that, I was working, and I had a sedentary job, although you get up and walk around, you know, but then I retired at Easter last year, so then I started doing walking groups”***(IDN13, female, aged 66, white British, pedometer only group, no increase).**

*“Because I work full time, you know, I go out, I walk the dog, umm, I’ve got a full time job, you know, and there’s not that many hours always to get that number of steps in.”***(IDN38, female, aged 59, white British, pedometer only group, increase).**

Having a pet was discussed by several participants as affecting their levels of physical activity:

*“We’ve got a dog now, so I do quite a lot of walking with her… She has to go out so we go out for quite long walks, especially at weekends, you know.”***(IDN20, male, aged 52, white British, nurse group, increase).**

### Routine

Participants spoke about their routine in relation to increasing their activity, identifying the influences of fixed or flexible routines and the emergence of new ones whilst participating in the trial.

Participants found that having a fixed, busy routine was often a barrier to increasing their walking due to the resulting time constraints. This was a particular problem for participants who were working in sedentary jobs or jobs that required long hours; it also included participants who were retired and reported established routines as a result of family roles or roles in their community:

*“I feel a bit … was letting myself down because I go to work very early in the morning and when I come back I’m totally tired… It would have been alright had, as I said, had I come back here, had different type of job I think, yes.”***(IDN27, male, aged 61, white and Asian pedometer only group, no increase).**

*“I do find it very difficult finding the time to do all that before so many other things I need to do, and look after my grandson, a three year old, it’s not easy, and I used to get really really tired…… obviously I could not keep up the you know the amount of steps every day. I’m not really sort of bothered as much how many steps I do, it’s how much time I can afford to go walking.”***(IDN17, female, aged 66, any other white background, pedometer only group, no increase).**

Other participants explained that the fluctuating nature of their routine could be a facilitator at times and a barrier at others. Some felt that this may have affected the trial outcome as they were not monitored during what they perceived to be a representative week at the 12-month follow up:

*“I have so much to do because even though I’m retired and that, I’m always doing something, and … depends what the day’s like to how much you can walk or you can you know take a long walk if you haven’t got an appointment somewhere or something like that, you know what I mean, so I could never quite reach that, but then again, at the end, I did.”***(IDN26, female, aged 65, white British, nurse group, no increase).**

*“I think the last week that I was monitored was a week when I didn’t do as many steps as perhaps I had some of the other weeks… it does fluctuate a bit I would say, but that particular week was less than I would have done normally in a week.”***(IDN16, female, aged 47, white British, nurse group, increase).**

A third group of participants spoke about a new routine which they had established with some attributing this directly to the trial and others feeling that they had been ready to make a lasting change towards healthier living anyway:

*“Since I’ve been doing this trial… my partner and I do walk the extra bus stop, I do walk more than I used to, so I am trying to do more walking since I’ve started this trial…I don’t walk as far as I’d like to, obviously, because in my daily life, as I said, I can’t do it, but I do walk … definitely I do walk more. If I hadn’t done this study I would most probably be getting on the bus at the bus stop at the bottom of my road.”***(IDN7, female, aged 60, white British, pedometer only group, no increase).**

### Monitoring

Monitoring, including both self-monitoring and external monitoring provided by the trial nurses, research assistants and trial equipment, was a broad theme mentioned by almost all participants. In most cases monitoring was perceived to be helpful and motivating but some participants discussed how failing to reach targets or not trusting the accuracy of the equipment could be a barrier.

Participants differed in their opinions regarding self-monitoring and targets, some suggesting they were already self-motivating, while others felt more empowered to self-monitor and self-motivate after completing the trial. Monitoring of behaviours and their outcomes were also important elements of the BCTs used in the trial (items 16 and 17 of Michie’staxonomy [15]):

*“Yes, setting my own targets and now, umm … well, it’s something that I’ve got used to now and I’m determined to keep it up.”***(IDN11, male, aged 70, white British, pedometer only group, increase).**

*“..well having something which counts the steps makes one conscious of it and filling out a little booklet every day, likewise, it just creates some personal pressure”***(IDN31, male, aged 59, white British, pedometer only group, no increase).**

Others valued the opportunity to discuss their progress with a nurse and felt this was an important factor in their progress. Participants reflected that the practice nurse was able to prompt review of behavioural goals, outcome goals and provide feedback on performance (items 10, 11 and 19 of Michie’staxonomy [15]):

*I think it was helpful seeing the nurse because it sort of made it more important to maybe think, oh well, oh gosh, I should have done that so I’d better do a bit more the next day. Not everybody might like that, but I found that quite helpful.”***(IDN35, female, aged 64, white British, nurse group, increase).**

*“..they kept saying how well I was doing, and all this sort of thing, so it made me want to continue. I think it was … a part motivation, yes, because I knew I had to face somebody and I didn’t want to fail.”***(IDN32, female, aged 63, white British, nurse group, increase).**

*“Very good, very helpful, it was really helpful, yes. Umm … I think, again, on that situation, it’s umm … it’s having someone to get some feedback from because then you know you aren’t doing things in vain.”***(IDN22, male, aged 63, white British, nurse group, no increase).**

However, the concept of rewarding oneself when a target has been met (item 12 of Michie’s taxonomy [15])was not frequently commented on by participants and those who did mention the use of rewards did not find them helpful:

“*It* [the handbook] *said things like, if you’ve reached your target this week, well done, haven’t you done well, treat yourself to a cup of tea. I found that rather condescending and umm … patronising. It just wrangled a bit … I don’t need a pat on the head and a piece of sugar”***(IDN41, male, aged 72, white British, pedometer only group, no increase).**

The monitoring provided by the trial equipment, particularly the pedometer, was a motivating factor for many participants although some doubted the accuracy of the pedometer and once they had ‘lost faith’ in the equipment its value as a monitoring device inevitably decreased:

*“I love the pedometer. I still use it. I’ll put it on one day, because it’s easy to forget, you know, sometimes you can only do like 6,000 or something, and if that is happening, I’ll maybe put it on for a couple of days then I’ll think, right, I’ve got to do a bit more. So it’s probably just to give me that motivation again.”***(IDN30, female, aged 51, white British, nurse group, increase).**

*“I gave up wearing the pedometer because I didn’t find that it registered the steps I was doing and, actually, I was quite disappointed when I first started wearing that because I thought, well, I’ve been walking for over an hour today and it had registered something like about 30 steps, and you think, well that’s obviously wrong, so I didn’t find the pedometer itself very useful and I soon gave up using that.”***(IDN39, female, aged 61, white British, nurse group, no increase).**

Setting goals and targets to increase the number of steps taken during the physical activity intervention was found to be both motivating and demoralising and many participants commented on this during their interview:

*“I think maybe because I just decided, after a few weeks, that it was unrealistic. I maybe just stopped trying at all rather than saying, you know, maybe it would have been better to say, okay, if it started off at 6,000, maybe I could have done 8,000 a day or something, but I just found 10,000 too much of a step up really to do, so I thought stop trying a little bit really.”***(IDN2, male, aged 45, white British, nurse group, no increase).**

*“..it was quite nice… having like a bit of a goal. When I was on the trial doing that amount of steps where you had the goals to achieve, which I thought would be quite easy, and sometimes it was like, oh crikey, how am I going to fit that in to the day or the week you know. But necessary I think so … although it was a bit of a nuisance, I think it was necessary.”***(IDN16, female, aged 47, white British, nurse group, increase).**

The importance of being set graded tasks (item 9 in Michie’s taxonomy [15]) was also identified by two participants:

*“I don’t know if it’s … was a nurse or more the programme as such, you know, how to stagger things and how to kind of like split them off, so yes, 30 minutes a day, but it could be 10 minutes, 10 minutes, 10 minutes. So that’s very useful I think.”***(IDN1, female, aged 48, any other white background, nurse group, increase).**

*“Psychologically, you know, to increase slowly over a period, and then keep a certain amount, is probably a good thing.”***(IDN3, male, aged 53, white British, pedometer only group, increase).**

### Support and social perspectives

Some participants reflected on the importance of nurse support, peer support, the support of friends and family and how their trial participation influenced those around them.

The support and encouragement provided by the nurse was greatly valued by participants, particularly with regard to identifying and overcoming barriers (item 8 of Michie’s taxonomy [15]) as was the importance of the support of the nurse or trial literature in providing advice on how, where and when to perform the behaviour changes (item 20 and 21 of Michie’s taxonomy [15]):

*“It’s something that I’ve never thought of because when I was talking, the complications, she says, no, no, just leave your car a distance, and then start walking, yes, she was very helpful.”***(IDN21, female, aged 47, black African, nurse group, increase).**

*“I suppose it’s just things like when you park the car, you know, park further away from where you would normally, maybe get off the train a stop early, that sort of thing.”***(IDN16, female, aged 47, white British, nurse group, increase).**

*“I think there was a lot of useful information in there* [the handbook] *because you had that, you know, like leaving your car and walking, which I did, and you know, different groups that you could join or going out with friends and, yes, there were lots of helpful suggestions and also I think, from what I remember, it signposted you to other websites and things.”***(IDN30, female, aged 51, white British, nurse group, increase).**

With any health behaviour change, relapse is an important risk. We looked for evidence of relapse prevention planning (item 35 of Michie’staxonomy [15]) and found a few examples where this had been mentioned and valued by participants, but interestingly the examples related to the trial handbook or equipment rather than the practice nurses:

*“Well the only thing I can think is you know sometimes if you let something slip and then it’s hard to get back into it, you know, if I’m perfectly honest, that has happened a couple of times where I’ve thought oh I can’t be bothered, but then, as it said in the booklet, which I think was good in the booklet, if you do find yourself slipping, don’t beat yourself up about it. You know, carry on and start again. I think, you know, if it’s … if you just keep that in mind, it’s a good approach.”***(IDN30, female, aged 51, white British, nurse group, increase).**

*“I think the booklet was good and the sort of comments, you know, about don’t get dispirited and things like that, I think that was excellent.. I think there was a point where they sort of said, you know, don’t give up now, or something like that, you know, at the point where … the novelty might have worn off…”***(IDN35, female, aged 64, white British, nurse group, increase).**

*“Definitely wearing the pedometer… I really do feel that’s kept us on the straight and narrow.”***(IDN18, female, aged 62, white British, nurse group, no increase).**

We specifically asked participants how they would feel about a similar trial which involved meeting with a nurse or facilitator in a group rather than individually. While some felt that this would be less desirable due to logistical and privacy considerations, many felt that they would have benefitted greatly from this and the possible benefits of meeting in a group also link to item 4 in Michie’s taxonomy [15] of providing normative information about others’ behaviour:

*“..if it involved each person reporting back on their success or failure at meeting the sort of previous targets, it might be a bit awkward in a group possibly”***(IDN2, male, aged 45, white British, nurse group, no increase).**

*“When you are with other people, and then you see the same problems they are facing, some of them might come up with other ideas… you know, we would meet the first day and we will see each other and then, if you want, you can form a team, support network, I don’t even know who else in my area was doing it.”***(IDN21, female, aged 47, black African, nurse group, increase).**

*“I think what would have been useful would have actually got some data on what the universe is doing you know, doing in terms of the highest step count, the lowest, the average, the mean..”***(IDN40, male, aged 48, white British, nurse group, no increase).**

*“And also reassurance, because I think, with the weather and that, she said that a few … I wasn’t alone in the fact that it had been a bit of a difficult keeping it going sort of thing, so that helps to know that.”***(IDN16, female, aged 47, white British, nurse group, increase).**

Meeting individuals whilst walking in their local area was described as being a benefit of trial participation for some participants:

*“..you know, it got me out in to the neighbourhood and both for my health but also socially, I was meeting other people, so that’s another positive thing.”***(IDN6, female, aged 51, white British, pedometer only group, no increase).**

Finally, some participants reflected on the beneficial effect that their trial involvement had on those around them, linking to item 29 of Michie’s taxonomy [15]), planning social support/social change:

*“…because my family and friends were aware, it was, you know, quite good because you’d sort of say shall we meet up and do this or go in to the nearest town but walk there rather than take the car, that type of thing.”***(IDN15, female, aged 49, any other white background, nurse group, no increase).**

*“…it's something I want to keep up, because I just felt that it was such a benefit, and even the kids would come out with me sometimes.”***(IDN30, female, aged 51, white British, nurse group, increase).**

## Discussion

### Principal findings

Perhaps most importantly, the trial was very well received by participants, with primary care thought to be a highly appropriate setting for such an intervention. A notable finding was that almost all participants that we interviewed felt they had succeeded in, and benefitted from, the intervention irrespective of their objective change in activity levels. These benefits appear to be both physical and psychological. Facilitators and barriers were generally common across both the nurse-led and the pedometer by post groups as well those who had increased and not increased their walking. Important facilitators included a desire for a healthy lifestyle, improved physical health, the enjoyment of walking in the local environment, having a flexible routine which allowed for an increase in walking, appropriate self and external monitoring and the support of friends, family and trial staff.

Important barriers included physical health problems, an inflexible routine not allowing for increased walking, work and other commitments, the weather and a mistrust of the monitoring equipment. There were a number of participants reporting significant health problems that both pre-dated the trial but also occurred during the trial, reflecting recruitment through primary care and the inclusion criteria that acknowledged that many chronic conditions are a reason to promote PA and not a reason to exclude from a PA intervention [16].

BCTs were variably alluded to by participants, but were mentioned considerably more frequently by those receiving the nurse intervention, with the exception of BCTs related to self-monitoring, which were mentioned much more frequently by the pedometer by post group. This suggests that the nurse support encouraged greater use of BCTs generally, but that the self-monitoring was particularly embraced by the postal group. Of the 22 elements that were specified in the trial protocol [16], those that were mentioned most often by participants and therefore seemed to have the most impact included: providing information about the link between behaviour and health; prompting self-monitoring and review of goals and outcomes; providing feedback; providing specific information about how, where and when to increase walking; planning social support/change; and relapse prevention. Use of rewards seemed to be the least helpful.

### Strengths and limitations

We recruited a large, balanced, purposive group of participants across six participating practices, including both men and women of different ages and different ethnicities and those who had both increased and not increased walking, from both intervention groups and which was not linked to a specific health condition or demographic group. We had an excellent response from those with whom we were able to make contact.

One to one in-depth discussions were undertaken with an interviewer not previously known to the participant,which may have allowed for a more candid exploration of barriers and facilitators and enhanced the credibility of our study, as did the involvement of a team of researchers in the data analysis and interpretation. Our interviews were conducted over a period of three months and our participants had received their interventions at different times of year which adds to the dependability of our findings. Although we were not successful in making contact with all participants who we telephoned, only one participant refused to be interviewed. This enhances the credibility and wider resonance of our study as it suggests that our participants broadly reflected those who took part in the overall trial thereby indicating that our findings may have wider transferability i.e. they may broadly applicable to other contexts.

As with all research, it is not possible to reach people who have not consented to participate at the outset, but this is not a major limitation of this study, which aimed to explore the experiences of trial participants in intervention arms. Although the majority of the interviews were conducted by one researcher this did not compromise the credibility of our findings. Our strategy of regular team meetings, the involvement of multiple researchers in the analysis and the testing of findings with the larger research group supported the credibility of our findings.

The trial within which this study was situated was conducted in an urban setting in South-West London, and so our findings are explicit to this particular context. This area is diverse in both ethnic and socio-economic terms, and therefore our findings may have wider resonance especially as we sought to ensure that our study included participants from a range of ethnic backgrounds which are under-represented in our trial and in research more broadly.

### Comparison with other studies

Many attempts have been made to elucidate the factors that promote physical activity adherence in adults and older adults and to understand barriers and facilitators to increasing activity in a trial setting [9, 28, 29]. However, this report is unusual in that it does not attempt to relate participants’ experiences to objective measures of success and, rather, seeks to understand the qualitative measures of success rather than the quantitative. Many of the barriers and facilitators that were identified by our participants have been reported in the literature previously thereby providing support for the credibility of our work. Barriers include poor physical health [19, 22–24, 27], lack of time [19, 23, 28], work and other family or home commitments [21, 28, 33–39] and the environment or weather [19, 22, 23, 27, 28]. The benefits of pedometer use to help set goals and step-count records to maintain accountability has been reported previously [40]. However, mistrust of the accuracy of monitoring equipment has not previously been reported as an important barrier, to our knowledge. Facilitators identified in the literature include social support [19, 22, 23, 26, 27, 29], self –efficacy [23–27]and a belief in the importance of physical activity for health [20, 22–24]. The importance of external monitoring and feedback, part of the Michie taxonomy [15], and reported by many of our participants, is not so prominent in the literature.

There are many examples in the literature, including systematic reviews and meta-analyses, of attempts to demonstrate which BCTs are most associated with self-efficacy and successful outcomes [41–43]. The following were all found to be positively associated with self-efficacy and/or an increase in PA: action planning [41, 42]; time management; prompting of self-monitoring; planning of social support/change [41]; provision of instruction; reinforcing effort towards behaviour [42]; tailoring; vicarious experience; feedback; and performing behaviour during the intervention [43]. The following were found to be negatively associated with self-efficacy: relapse prevention [42]; setting graded tasks [42, 43]; persuasion; and barrier identification [43]. In agreement with the existing literature, our study suggests that prompting self-monitoring and review of goals and outcomes; providing feedback and planning social support/change were well received BCTs. In addition, our participants reported appreciation of receiving information about the link between behaviour and health and specific information about how, where and when to increase walking. In contrast to the literature, several of our participants reported finding relapse prevention techniques to be helpful. Use of rewards was found to be unhelpful by the participants who mentioned this BCT in our study; however, this BCT was not found to be either positively or negatively associated with self-efficacy in the existing literature [41–43].

## Conclusions

Participants’ experiences are shaped both by factors outside of the control of the trial team (such as work commitments and physical health) and within the control of the trial team (such as trial equipment and providing feedback), thus suggesting areas on which future trialists can focus their efforts.

Participants valued information about the benefits of an intervention and also how they compare with others, both elements which could be readily incorporated into such interventions or programmes. Many participants want to be supported, be it by family, friends or trial staff, while others prefer the option to be more self-directed and self-efficacious. This suggests that a tailored programme, where participants can opt for different levels of support from trial or programme staff, might be well received. Future research could focus more specifically on which BCTs are most valued by participants and perhaps how their responsiveness correlates with their objective success.

We have clearly demonstrated that there is more than one way to measure the success of a complex intervention and the benefits enjoyed by participants may extend well beyond the objective outcomes. We chose to define an increase as >/=200 steps/day but would speculate that a higher threshold may have produced results in which the narratives differed more markedly between those who increased their PA and those who did not. This could be an area for further exploration in future research.

The prominence of health conditions among our participants, for whom this intervention is particularly important, reinforces the value of the primary care setting where there will be ready access to advice. Investing further in such interventions in a primary care setting would seem justified by our findings.

## Appendix 1

*Example schedule for patient in the nurse group who increased walking; minor changes were made for patients in pedometer only group or who did not increase activity.*

### Opening questions

1. Firstly, I’d like to hear about your experience of participating in the PACE-UP exercise trial?
  - What did you like or not like about being involved in the trial?
  - What was it about x/y that you liked or didn’t like?
2. Do you think that your feedback sheet accurately reflects your activity at these time points over the trial? If not, why not?
3. How did you manage to increase your activity levels? Can you tell me more about that?
4. Do you feel that you are maintaining your increased activity levels? Why/why not?
5. Would you say that your progress has been influenced by the weather or season?
  - In what way has it influenced your progress?
6. How did you feel about your step count targets?

### Nurse questions

1. Tell me about your experiences meeting with the nurse?
2. Do you think meeting the nurse was a factor in you increasing your activity?
3. Did you find the nurse approachable/understanding of your concerns and goals? Why/why not?
4. Did the nurse help you to develop your own strategies and goals? Did you adapt the strategies and goals yourself?
5. Sometimes it can be difficult to maintain your progress with physical activity. Did you find the nurse helpful if/when you were finding maintaining your progress difficult? Why/why not?
6. Thinking about the recommendations and suggestions made by the nurse:
  - Did any stand out for you as particularly helpful? Why?
  - Did any stand out for you as unhelpful? Why?
7. With regard to your meetings with the nurse, was there anything that came out of those meetings that you didn’t expect or was a surprise to you?
8. Did you think the number and length of the sessions was about right/too much/not enough? Why?
9. What did you think about the location for the meeting, in your practice?
  - Appropriate?
  - Convenient?
  - Would you have preferred somewhere else? If so, where?

### Materials and equipment

1. Trial equipment –
  - How did you find wearing the belt with the chunky pedometer and accelerometer at baseline? (and the belt and accelerometer at 3 and 12 months)
  - Was it comfortable? Why/why not?
  - Did you have difficulties with your clothes when wearing the belt? Can you tell me more about this?
  - Did you have any problems remembering to wear the equipment? How did you overcome these problems?
2. We asked you to keep a detailed diary of your activity at the beginning of the exercise programme. How did you find this? Can you tell me more?
3. Handbook and diary
  - How did you use the handbook and diary?
  - Was it useful/relevant to you? Why/why not?
  - Was the handbook useful if/when you were struggling to maintain your progress? Why/why not?
  - Was keeping a diary motivating? Why/why not?
  - Any suggestions or improvements for these materials?
4. Pedometer
  - What was your experience of using the pedometer? Did you have any problems operating it? How did you overcome these problems?
  - Did you find the nurse helpful in understanding how to operate it? Why/why not?
  - Did you find getting your step count feedback from the pedometer was helpful? Why/why not?
  - Are you still using it? Why/why not? (if broken, we can offer to send a new one!)

### Other questions

1. Were there any factors outside the study that helped or hindered you to increase your activity?
  - Did you get any support from friends or family? How did the support (or lack of) affect you progress?
  - Did you join a walking group? How did this affect your progress?
2. **Only for couples:** Did having another person in the household also taking part in the study help or hinder your progress? Can you tell me more about this?
3. Some people like to work individually and others prefer to work in a group. If PACE-UP involved meeting in a group instead of one to one with a nurse, would you have been more or less likely to take part? Why/why not?
4. Have you made any changes to your routine or life as a result of being part of the study?
  - How do you plan to maintain any changes that you’ve made?
  - Do you foresee any problems or barriers?
5. Some people have suggested that it is more difficult to keep going when you are not feeding back to anyone. Do you think that knowing you would be feeding back your progress at 12 months would have been a useful motivating factor?
6. Finally – any suggestions about how we could have changed or improved the exercise programme?
